# An Adjusted Probability Method for the Identification of Sociometric Status in Classrooms

**DOI:** 10.3389/fpsyg.2017.01836

**Published:** 2017-10-18

**Authors:** Francisco J. García Bacete, Antonius H. N. Cillessen

**Affiliations:** ^1^Department of Developmental, Educational and Social Psychology and Methodology, GREI Interuniversitary Research Group, Universitat Jaume I, Castellón de la Plana, Spain; ^2^Behavioural Science Institute, Radboud University, Nijmegen, Netherlands

**Keywords:** sociometric status, peer nominations, peer relationships, neglected status, elementary school

## Abstract

**Objective:** The aim of this study was to test the performance of an adjusted probability method for sociometric classification proposed by García Bacete (GB) in comparison with two previous methods. Specific goals were to examine the overall agreement between methods, the behavioral correlates of each sociometric group, the sources for discrepant classifications between methods, the behavioral profiles of discrepant and consistent cases between methods, and age differences.

**Method:** We compared the GB adjusted probability method with the standard score model proposed by Coie and Dodge (CD) and the probability score model proposed by Newcomb and Bukowski (NB). The GB method is an adaptation of the NB method, cutoff scores are derived from the distribution of raw liked most and liked least scores in each classroom instead of using fixed and absolute scores as does NB method. The criteria for neglected status are also modified by the GB method. Participants were 569 children (45% girls) from 23 elementary school classrooms (13 Grades 1–2, 10 Grades 5–6).

**Results:** We found agreement as well as differences between the three methods. The CD method yielded discrepancies in the classifications because of its dependence on *z*-scores and composite dimensions. The NB method was less optimal in the validation of the behavioral characteristics of the sociometric groups, because of its fixed cutoffs for identifying preferred, rejected, and controversial children, and not differentiating between positive and negative nominations for neglected children. The GB method addressed some of the limitations of the other two methods. It improved the classified of neglected students, as well as discrepant cases of the preferred, rejected, and controversial groups. Agreement between methods was higher with the oldest children.

**Conclusion:** GB is a valid sociometric method as evidences by the behavior profiles of the sociometric status groups identified with this method.

## Introduction

The fact that social status in the peer group predicts youths' future adjustment has led to great interest in sociometric methods (e.g., Cillessen and Bukowski, 2017). The most common method for the assessment of sociometric status is peer nominations, in which children or adolescents nominate others from their peer group for certain criteria (Poulin and Dishion, 2008; Cillessen, 2009; Hymel et al., 2010). Peer nominations are a relatively simple technique because they are easy to implement and easy to understand for participants (Gommans and Cillessen, 2015).

Cillessen and Bukowski (2000) pointed to three critical events in the history of sociometric methods for the study of peer relations: (a) the change from one-dimensional to two-dimensional systems (the use of both positive and negative nominations), (b) the introduction of two independent composite dimensions, social preference (acceptance minus rejection), and social impact (acceptance plus rejection), and (c) the identification of the five sociometric groups or types (preferred, rejected, neglected, controversial, and average; Coie et al., 1982); in this paper we use *preferred* rather than *popular*, since popularity has a different meaning, see Cillessen and Marks (2011). Two main scoring systems have been used for the classification of sociometric groups: the standard score model that assigns children to groups based on *z*-scores (Coie et al., 1982), and the probability model, that makes assignments based on the probability of scores (Newcomb and Bukowski, 1983).

The extensive literature on the correlates of sociometric status provides evidence for the validity of the sociometric groups (e.g., Bierman, 2004; Asher and McDonald, 2009), in particular the accepted and rejected groups. The meta-analysis by Newcomb et al. (1993) demonstrated that children of each type have a unique behavioral repertoire: preferred children are sociable, but not aggressive or withdrawn; rejected children are aggressive or withdrawn, but not sociable; neglected children are low in aggression and sociability; and controversial children score high on both (see also García Bacete, 2006).

Some researchers have used ratings instead of nominations (e.g., Ortíz et al., 2002; Maassen et al., 2005) but ratings are less amenable to the identification of sociometric groups (Bukowski et al., 2000). In the practice of sociometric research, there is still great variability in the methods used for sociometric classifications (see e.g., Martin, 2011; Aparisi et al., 2015). Therefore, it is still useful to compare the strengths and weaknesses of different classification systems. Previous comparisons have not been conclusive about which method is “best” (Newcomb and Bukowski, 1983; Terry and Coie, 1991; Frederickson and Furnham, 1998; Chan and Mpofu, 2001; Maassen et al., 2005; Mckwon et al., 2011).

Our overall purpose was to compare two classification procedures, standard score method (CD; Coie and Dodge, 1983), and probability score method (NB; Newcomb and Bukowski, 1983). As novelty, we introduce a new probability method (GB; García Bacete, 2006), that adjusts to the raw scores of each classroom and proposes a new criterion for the classification of the neglected students.

### Standardization and probabilistic methods

Although the standard score procedure facilitates comparisons of findings between studies, it has certain limitations, such as the assumed similarity between standardized and absolute scores and the assumed independence of the dimensions social preference and social impact (Newcomb and Bukowski, 1983; Maassen et al., 2005).

Bronfenbrenner (1945) indicated that procedures based on *z*-scores depend on the standard deviation and range of nominations in the group. According to Newcomb and Bukowski (1983), the standard score method can lead to, but does not guarantee, similarity with the raw scores. The use of *z-*scores results in the similar percentages of each category in all classrooms, which may not actually be the case (Maassen et al., 2005). *Z*-scores also make it difficult to study long-term development or the effects of an intervention, because they do not reflect changes due to context, voter population, or intervention (Maassen et al., 2005). Likewise, Newcomb and Bukowski (1983) stated that Coie et al.'s (1982) procedure was problematic in peer groups with a well-integrated social network in which few children are disliked by everyone and likings are evenly distributed among peers.

The standard score classification system is based on social preference and impact as independent dimensions to distinguish groups (Coie et al., 1982; Maassen et al., 2005). To begin with, we may recall that social preference and impact are composite dimensions of positive and negative nominations received, but the fact that the distributions of acceptance and rejection are different (Gommans and Cillessen, 2015) makes it questionable whether they can simply be added or subtracted from one another. Besides, in the standard score model, preference, and impact are independent because of the way they are calculated, but, as Bukowski et al. (2000) showed, the association between preference and impact may be curvilinear. For example, children who are highly accepted are usually low in rejection, but poorly accepted children may vary in their level of rejection.

Because of these limitations, Newcomb and Bukowski (1983) (NB) used binomial probabilities of raw “liked most” and “liked least” scores to provide a reference system for sociometric choices. This procedure uses two dimensions but it does not combine them, except for the classification of neglected students. However, their system uses fixed cutoffs for each group, making it difficult to solve some of the limitations mentioned above. To address this issue, García Bacete (2006) (GB) suggested using the binomial probability to calculate the cutoffs (rare scores not expected by chance) for each classroom.

In addition to the differences based on the use of raw scores or *z*-scores and of two single dimensions or two composite dimensions, the methods also differ in the criteria and cutoffs proposed, whether these are fixed or can adjust to the characteristics of each classroom. The criteria mainly, but also the cutoffs, concern issues such as which percentage of students classified in each sociometric group is expected for each method. For example, a percentage around 15% for each sociometric group by CD, 5% by NB, while GB percentage is in an intermediate position. For the sake of clarity, we addressed the comparison between methods of the specific criteria and cutoffs in the method section, while the questions regarding *z*-scores and composite dimensions, which are a core issue for the differences between standardized and probability methods, are examined in the results section because we used data to illustrate these questions.

Comparative studies have examined the percentage of children in each sociometric group, the agreement between methods, and the concurrent validity of each group identified by each method. Overall, about 10–15% is preferred, the same percentage is rejected, about 12–17% is neglected, and about 2–6% is controversial (Newcomb et al., 1993), but the group percentages varied depending on the method used (Terry and Coie, 1991). Agreement between methods were usually moderate (Terry and Coie, 1991; Frederickson and Furnham, 1998). Furthermore, Frederickson and Furnham (1998) pointed out that similar distributions of types by the different methods in a sample do not necessarily lead to high levels of agreement in allocating particular subjects to categories. This makes it necessary to identify the discrepant cases and analyze the sources of disagreement (Maassen et al., 2005).

### Identification of the neglected group

The difficulty correctly identifying neglected students has been repeatedly noticed (Newcomb et al., 1993; Frederickson and Furnham, 1998; Maassen et al., 2005; García Bacete, 2006). Therefore, Coie and Dodge (1983) (CD) replaced the original strict criterion of receiving no positive nominations in their standard score model (Coie et al., 1982) with the criterion that both positive and negative nominations be below the classroom mean.

For the same reason, García Bacete (2006) (GB) modified the criterion of NB for neglected students (positive nominations + negative nominations ≤2). Contrary to CD, GB suggested that the rate of the neglected group might be lower and that we should differentiate between positive and negative nominations. They proposed that the cutoff for positive nominations should be 1 and for negative nominations less than the mean, to maximize the difference with average students. García Bacete (2006) identified 10.7% neglected students (compared to 13.2% by NB and 14% by CD); 92.3% of these neglected students were well-classified according to behavioral correlates (compared to 87.5% in NB and 70.6% in CD).

### Present study

This study had four goals. Goal 1 was to examine the similarities and differences between the three methods in terms of overall agreement and agreement by type. We expected moderate to high agreement between methods. Goal 2 was to examine whether the sociometric classification by each method was supported by the behavioral correlates. We expected that the results would confirm the behavioral differences between the sociometric types. Goal 3 was to examine the classification criteria and cutoffs proposed by each method, the use of *z*-scores or raw scores and of two single or two composite dimensions, as sources that could explain the disagreement between methods. We expected to identify a number of elements that influence the classification and account for the associations between these elements and the classification. Goal 4 was to examine the characteristics of students who were classified differently by the different methods (discrepant or inconsistent cases), and compare them with students who were classified identically by the different methods (consistent cases), in particular the neglected students. We expected the inconsistent cases to be behaviorally atypical. The behaviors of the discrepant cases classified by GB, in comparison with the other methods, will not differ from the behaviors of the consistent cases. More specially, the GB method will perform better to classify neglected students. As data were collected at the beginning of elementary school (Grades 1–2) and at the end (Grades 5–6), it was also possible to examine age differences. In general, we expected the agreement between methods to be higher with older children.

## Method

### Participants and procedure

Sample 1 consisted of 226 children (46.9% girls) from 10 classrooms (range 16–26 children per class) at the beginning of elementary school (Grades 1–2) of five public schools in Spain. Sample 2 consists of 343 children (44.3% girls) from 13 classrooms (range 17–35 children per class) at the end of elementary school (Grades 5–6) of four public schools in Spain. Schools were selected randomly from all schools in their district and representative of medium socioeconomic status. Because of the excellent collaboration of the schools, the participation rate was 100% in all classrooms except three, in which the participation rates were 88, 96, and 97%. For data collection, we interviewed all the children of sample 1 individually in a quiet room at their schools to ensure comprehension. Sample 2 children answered the questionnaires in their classrooms.

#### Ethics statement

The present study was conducted in accordance with the 1964 Helsinki declaration and its later amendments, with the approval of the management board of schools and the educational inspection services. The study was reviewed and approved by the Professional Ethics Committee of Universitat Jaume I. Participation in the study was voluntary and written and informed consent was obtained from the parents/legal guardians of all participants.

### Measures

#### Measures of social behavior

Classroom behavior was assessed with the Pupil Evaluation Inventory (PEI; Pekarik et al., 1976), validated for Spanish samples by Villanueva (1998). We asked children in Grades 5–6 to nominate classmates who best fit each of 34 descriptions. The items represented three factors: *aggression* (20 items, 37.6% variance; e.g., starts fights, gets into trouble); *withdrawal* (9 items; 15.7% variance; e.g., someone who is often left out, shy); and *likeability* (5 items; 7.5% variance; e.g., liked by everyone, best friend). For Grades 1–2 we used a short version of 17 items developed by Villanueva et al. (2001), that reproduced the factorial distribution of the PEI complete scale (9, 5, and 3 items respectively). Shortened versions of the PEI have effectively discriminated sociometric groups in previous studies (e.g., Wrobel et al., 2005). Three total scores for each child were obtained by summing all his/her frequencies in the items that composed each factor. These scores were standardized within classrooms. Cronbach's α was above 0.70 for all scales.

#### Sociometric nominations

We asked students to name three peers in their classroom “whom they liked most” and “whom they liked least.” These items have shown adequate test-retest reliability and convergent validity (Cillessen, 2009). Nominations were limited to three because the NB method only offered criteria under this condition. For every student, the number of positive and of negative nominations received (PNR and NNR) were counted, thus generating a *pair* of raw scores for each student (PNR, NNR). Each student was therefore characterized by his/her pair of sociometric scores. We examined then which pairs were classified in each sociometric type by each method. We explained below the classification criteria used by each sociometric method.

##### Standard score method

In the standard score method (Coie et al., 1982), the raw numbers of PNR and NNR are standardized within classrooms to standard scores for “liked most” (Z_PNR_) and “liked least” (Z_NNR_). Social impact (SI = Z_PNR_ + Z_NNR_) and social preference (SP = Z_PNR_-Z_NNR_) are calculated and again standardized (Z_SI_ and Z_SP_) within classrooms. Criteria for the five sociometric types were: preferred Z_SP_ > 1, Z_PNR_ > 0, and Z_NNR_ < 0; rejected Z_SP_ < −1, Z_PNR_ < 0, and Z_NNR_ > 0; neglected Z_SI_ < −1, and PNR = 0; controversial Z_SI_ > 1, and Z_PNR_ and Z_NNR_ > 0; and average Z_SP_ and Z_SI_ between −0.5 and 0.5. The remaining participants had no classification. Coie and Dodge (1983) (CD) modified this system in order to classify of all children. In the revision, the average group consisted of all children who did not meet the criteria for the first four groups. They also changed the criterion for the neglected group (Z_SI_ < −1, and Z_PNR_ and Z_NNR_ < 0), making it larger. In this study, we used this modified version by Coie and Dodge (1983).

##### Probability method NB

The NB method is based on binomial probabilities computed for each number of nominations received. This number is considered “rare” if it falls beyond chance level. A child is preferred when PNR ≥ 7 and NNR < *M*_NNR_ (mean of NNR); rejected when NNR ≥ 7 and PNR < *M*_PNR_ (mean of PNR); neglected when SI_raw_ = (PNR + NNR) ≤ 2; controversial when either [PNR ≥ 7 and NNR ≥ *M*_NNR_] or [NNR ≥ 7 and PNR ≥ *M*_PNR_]; the remaining participants are classified as average. These criteria are valid for groups of 13–50 participants, with nominations limited to 3, and a probability level of 0.05.

##### Probability method GB

The GB method is an adaptation of the NB method. The fundamental changes suggested by GB are: (1) to calculate the cutoffs for each of the sociometric status groups in each classroom studied, instead of using fixed and absolute cutoffs; (2) to propose a new criterion for the identification of neglected students; (3) to extend the use of the proposed criteria to limited and unlimited nominations. PNR and NNR are analyzed by calculating continuous binomial probabilities using Salvosa's tables. Based on *t*-values and a probability level of 0.05, upper and lower limits can be set for positive nominations (UL_PNR_ and LL_PNR_) and negative nominations (UL_NNR_ and LL_NNR_) for groups of a certain size. Calculations are made with the *Sociomet* software (González and García Bacete, 2010). A child is classified as preferred when PNR ≥ UL_PNR_ and NNR < *M*_NNR_; rejected when NNR ≥ UL_NNR_ and PNR < *M*_PNR_; neglected when PNR ≤ 1 (in case of 5 or unlimited nominations the value should be the largest value of LL_PNR_ or 1) and NNR < *M*_NNR_; controversial when either [PNR ≥ UL_NPR_ and NNR ≥ *M*_NNR_] or [NNR ≥ UL_NNR_ and PNR ≥ *M*_PNR_]; the remaining participants are classified as average. This method has shown excellent discriminant validity in a sample of Grade 4 children (García Bacete, 2006).

##### Comparison of criteria and cutoffs

We examined first the cases of preferred, rejected and controversial students and then the case of neglected students. For preferred, rejected, and controversial students, the probability methods have two criteria: a specific one, which is a cutoff score that must be exceeded in one dimension, and a supplementary one, which is not exceeding the mean of the other dimension (for controversial students the mean of the other dimension must also be exceeded). We focused on the specific criterion.

The NB procedure simplifies criteria by proposing upper limits fixed at seven nominations for both PNR and NNR. This limit appears to be high. It is assumed to be associated with a *p*-value of 0.05, but when the number of voters is not the maximum the *p*-value may be less than 0.01. In GB method, upper limits are calculated for each classroom, one for PNR and one for NNR. The upper limits depend on the average number of nominations given by the voters. They are associated with a *p*-value of 0.05; nevertheless, since the raw nominations are whole numbers, in many classrooms the *p*-value become more restrictive, although not as much as in NB.

The CD uses three *z*-score criteria, one specific and two supplementary. As specific criterion, CD uses a standardized score for the composite dimensions SP (Z_SP_) and SI (Z_SI_). For preferred students, Z_SP_ > 1 (supplementary criteria: Z_PNR_ > 0 and Z_NNR_ < 0); for rejected students, Z_SP_ < −1 (supplementary criteria: Z_PNR_ < 0 and Z_NNR_ > 0, and Z_SI_ > 1); for controversial students, Z_SI_ > 1 (supplementary criteria: Z_PNR_ > 0 and Z_NNR_ > 0). CD uses fixed cutoffs, as does NB, hence it does not adjust to each classroom. The cut-off of ± 1*z* leads to percentages around 15% for each type in all classrooms. Moreover, in CD, unlike in the probability procedures, PNR and NNR are complementary, only ensuring minimum distance between both in the case of the preferred and rejected children and being interchangeable in the case of the controversial and neglected children. Hence, students with low scores may be classified as preferred, rejected, or controversial. Furthermore, since it uses *z*-scores, small variations in *z*-scores may lead to changes in the classification of participants.

For neglected students, NB also uses a criterion with a fixed cutoff for a composite dimension: SI_raw_ = (PNR + NNR) ≤ 2. In this case, NB imposes a dependency between PNR and NNR, and it does not differentiate between PNR and NNR. According to this criterion, six *pairs* of nominations (PNR and NNR) between *pair* 2-0 and *pair* 0-2 are possible for the neglected group in NB (2-0, 1-1, 1-0, 0-0, 0-1, and 0-2). Regarding GB, it does not use a composite dimension of PNR and NNR. GB applies the criteria of not receiving more than 1 PNR and being situated below the *M*_NNR_. Like NB, it also allows 6 *pairs* of nominations, but, as GB seeks for greater differences between neglected and average, on the one hand it is more restrictive with the positive nominations (*pair* 2-0 is not allowed for neglected students) and on the other hand it allows the *pair* 1-2. The possible *pairs* for neglected in GB are: 1-1, 1-0, 0-0, 0-1, 0-2, and 1-2.

The CD procedure requires that Z_SI_ < −1 and that both PNR and NNR be below the mean. These criteria allow the 8 possible *pairs* between 2-1 and 1-2. In practice, this comes down to using SI_raw_ ≤ 3, being less restrictive than the other two methods. CD is the only procedure that allows the *pair* 2-1. For this criterion also applies what we above mentioned about the other types classified by CD.

## Results

### Research question 1: agreement between procedures

#### Distribution of sociometric types

Table 1 shows the distribution of the sociometric groups for each method. Percentages were 14.6 and 6.2% for the preferred group, 15.5 and 6.1% for the rejected group, 4.9 and 1.3% for the controversial group, and 12.3 and 18.7% for the neglected group, according to CD and NB, respectively. The percentages for GB were in between the percentages for CD and NB for each group.

**Table 1:** Number and percentage of students in each sociometric group by method.

	**Preferred *N* (%)**	**Rejected *N* (%)**	**Neglected *N* (%)**	**Controversial *N* (%)**	**Average *N* (%)**	***k* CD-NB**	***k* CD-GB**	***k* NB-GB**
**SAMPLE 1**								
CD	33 (14.6)	35 (15.5)	39 (17.3)	11 (4.9)	108 (45.3)			
NB	14 (6.2)	14 (6.1)	39 (17.3)	3 (1.3)	156 (69.0)	0.57	0.66	0.62
GB	23 (10.2)	29 (12.8)	28 (12.4)	6 (2.7)	140 (61.9)			
**SAMPLE 2**								
CD	44 (12.9)	48 (14.0)	42 (12.3)	20 (5.8)	188 (55.0)			
NB	24 (7.0)	26 (7.6)	64 (18.7)	10 (2.9)	218 (63.7)	0.61	0.78	0.63
GB	39 (11.4)	41 (12.0)	47 (13.7)	17 (5.0)	198 (57.9)			

*CD, Coie and Dodge method (1983); NB, Newcomb and Bukowski method (1983); GB, García Bacete method (2006); k, Cohen's kappa score*.

#### Overall agreement between methods

Agreement between classifications was determined with Cohen's kappa test, following Landis and Koch's (1977) guide to interpret the *k* value (*k*). In Sample 1, overall agreement between methods was moderate (*k* = 0.57 for CD-NB) to good (*k* = 0.66 for CD-GB and *k* = 0.62 for NB-GB). In Sample 2, overall agreement between the three methods was higher than in the Sample 1, especially for GB (*k* = 0.78 with CD, *k* = 0.63 with NB). Kappa value for CD-NB was 0.61.

#### Agreement between methods for each sociometric group

Table 2 presents the number of participants identified, the number of agreements, and Cohen's kappa between methods for each group. For the preferred group, kappa ranged from 0.56 to 0.85. For the rejected group, kappa ranged from 0.48 to 0.81. For the controversial group, agreement was moderate in Sample 1 (0.42 to 0.66) and good in Sample 2 (0.66 to 0.86). For the neglected group, agreement between methods was higher, ranging from 0.60 to 0.85.

**Table 2:** Agreement between procedures for each sociometric type and number of students for each procedure.

		**Sample 1**			**Sample 2**		
		**CD/NB**	**GB/CD**	**GB/NB**	**CD/NB**	**GB/CD**	**GB/NB**
Preferred	*k*	0.56	0.80	0.74	0.68	0.85	0.74
	N	33/14/**14**-*33*	23/33/**23**-*33*	23/14/**14**-*23*	44/24/**24**-*44*	39/44/**36**-*47*	39/24/**24**-*30*
Rejected	*k*	0.48	0.67	0.62	0.64	0.81	0.75
	N	35/14/**13**-*36*	29/35/**23**-*41*	29/14/**14**-*29*	48/26/**25**-*49*	41/48/**37**-*52*	41/26/**26**-*41*
Neglected	*k*	0.85	0.74	0.67	0.69	0.78	0.61
	N	39/39/**34**-*44*	28/39/**26**-*41*	28/39/**24**-*43*	42/64/**39**-*75*	47/42/**36**-*53*	47/64/**37**-*74*
Controversial	*k*	0.42	0.57	0.66	0.65	0.86	0.73
	N	11/3/**3**-*11*	6/11/**5**-*12*	6/3/**3**-*6*	20/10/**10**-*20*	17/20/**16**-*21*	17/10/**10**-*17*

*CD, Coie and Dodge method (1983); NB, Newcomb and Bukowski method (1983); GB, García Bacete method (2006)*.

*k, Cohen's kappa score*.

*xx/yy/**aa**-ss. xx, students classified by first method; yy, students classified by second method; **aa**, students classified by both method (number of agreements in **bold**); ss, students classified by only one of the two method (in italics)*.

The percentages of students identified by any procedure were in Sample 1 and 2 respectively, 15 and 14% (preferred), 19 and 15% (rejected), 5 and 6% (controversial), and 20 and 22% (neglected). The three procedures agreed on 47% of preferred students, 40% of rejected students, 40% of controversial students, and 48% of neglected students.

All preferred, rejected, and controversial students classified by NB were classified the same by GB and CD, and most classified by GB were also by CD. CD had the highest disagreements with one of the other methods (102: 39 preferred, 45 rejected, 18 controversial) or with both methods (50: 17 preferred, 23 rejected, 18 controversial). For the neglected group, NB identified more neglected students than GB, and GB more than CD. However, there was variability, as 63 neglected students were classified differently by another method. There were 38 disagreements between CD and NB, 32 between CD and GB, and 56 between NB and GB.

### Research question 2: behavioral correlates for each type in each method

We tested whether the sociometric classification was supported by a behavioral basis, that is, whether the sociometric groups identified by each method presented differences in their social behavior. To this end, in each one of the two samples and for each classification method, we conducted three one-way ANOVA—for aggression, withdrawal, and likeability—with the five sociometric types as factor. We applied the Bonferroni correction to the initial *p*-value (*p* < 0.05), requiring then *p*-value < 0.017. In the *post-hoc* multiple comparisons, we selected Tukey's HSD test when the data met the homogeneity of variances assumption, and Games Howell test when they did not. Table 3 shows the results.

**Table 3:** ANOVAs for the standardized social behavior scores.

	**Preferred**			**Rejected**			**Neglected**			**Controversial**			**Average**			**ANOVA**		***Post-hoc*** **comparisons**									
	***N***	**M**	***SD***	***N***	**M**	***SD***	***N***	**M**	***SD***	***N***	**M**	***SD***	***N***	**M**	***SD***	***F***	***p***	**P-R**	**P-N**	**P-C**	**P-A**	**R-N**	**R-C**	**R-A**	**N-C**	**N-A**	**C-A**
**SAMPLE 1**																											
**Aggression**																											
CD	33	−0.30	0.52	35	0.81	1.38	39	−0.42	0.68	11	0.79	1.30	108	−0.10	0.78	12.3	0.000	0.001				0.000		0.005			
NB	14	−0.22	0.63	14	1.5	1.52	39	−0.35	0.72	3	2.25	1.51	156	−0.06	0.82	17.8	0.000	0.009				0.004		0.009			
GB	23	−0.30	0.50	29	0.99	1.37	28	−0.31	0.77	6	1.27	1.45	140	−0.15	0.78	15	0.000	0.000				0.001		0.001			
**Withdrawal**																											
CD	33	−0.38	0.40	35	0.74	1.37	39	−0.16	0.76	11	0.21	1.45	108	−0.09	0.86	6.6	0.000	0.000			0.049	0.010		0.014			
NB	14	−0.40	0.37	14	1.4	1.40	39	−0.12	0.79	3	−0.28	0.78	156	−0.05	0.91	9.2	0.000	0.004				0.016		0.020			
GB	23	−0.47	0.4	29	0.66	1.3	28	−0.16	0.85	6	0.46	1.75	140	−0.05	0.88	5.7	0.000	0.001			0.004	0.049		0.060			
**Likeability**																											
CD	33	1.22	1.18	35	−0.58	0.42	39	−0.33	0.57	11	−0.10	0.88	108	−0.04	0.86	16.5	0.000	0.000	0.000	0.006	0.000			0.000			
NB	14	1.41	1.2	14	−0.65	0.50	39	−0.37	0.56	3	−0.33	−40	156	0.03	0.95	12.6	0.000	0.000	0.001	0.008	0.006			0.002		0.007	
GB	23	1.3	1.21	29	−0.55	0.46	28	−0.47	0.55	6	−0.53	0.37	140	0.016	0.90	19.4	0.000	0.000	0.000	0.000	0.000			0.000		0.003	0.065
**SAMPLE 2**																											
**Aggression**																											
CD	44	−0.32	0.65	48	0.97	1.52	42	−0.43	0.3	20	0.93	1.17	189	−0.18	0.70	21.9	0.000	0.000		0.001		0.000		0.000	0.001	0.004	0.004
NB	24	−0.28	0.74	26	1.13	1.73	64	−0.42	0.31	10	1.13	1.31	219	−0.03	0.85	18.9	0.000	0.005		0.051		0.001		0.018	0.028	0.000	
GB	39	−0.32	0.66	41	0.95	1.53	47	−0.35	0.36	17	0.85	1.21	199	−0.12	0.80	19.8	0.000	0.000		0.013		0.000		0.001	0.007	0.032	0.033
**Withdrawal**																											
CD	44	−0.41	0.36	48	0.87	1.4	42	0.16	1.17	20	0.048	1.02	189	−0.17	0.74	15.5	0.000	0.000	0.028		0.013			0.000			
NB	24	−0.39	0.41	26	0.85	1.4	64	0.07	1.05	10	0.42	1.3	219	−0.10	0.87	7.55	0.000	0.001	0.029		0.052			0.016			
GB	39	−0.41	0.36	41	0.76	1.32	47	0.14	1.12	17	0.29	1.3	199	−0.13	0.82	10.4	0.000	0.000	0.020		0.009			0.001			
**Likeability**																											
CD	44	1.06	1.21	48	−0.59	0.30	42	−0.38	0.80	20	0.34	1.01	189	−0.05	0.86	18.8	0.000	0.000	0.000		0.000		0.005	0.000			
NB	24	1.4	1.28	26	−0.55	0.32	64	−0.16	1.06	10	0.19	0.94	219	−0.04	0.84	17.4	0.000	0.000	0.000	0.046	0.000			0.000			
GB	39	1.1	1.25	41	−0.56	0.38	47	−0.33	0.9	17	0.24	1.09	199	−0.04	0.84	21.6	0.000	0.000	0.000		0.000		0.050	0.000			

*CD, Coie and Dodge method (1983); NB, Newcomb and Bukowski method (1983); GB., García Bacete method (2006). Only the significant comparisons are presented (the level of significance can be found in the Table)*.

Across the three methods, 85 group differences were significant, 47% of all possible comparisons (42% of comparisons in Sample 1; 53% in Sample 2). The three methods agreed on the behavioral differences between the groups, especially for comparisons involving rejected (65%) or preferred (63%). The differences in social behavior according to sociometric type observed in each method were similar in the two samples. The three procedures coincided in their difficulty to establish behavioral differences between the controversial group and the other groups (only 21% of the comparisons).

GB found more behavioral differences across all types (50%) than the other two methods (47% for CD, 45% for NB). When there were discrepancies between methods, NB was usually involved (in 4 of the 6 discrepancies identified). NB had more difficulties than CD and GB to differentiate the average group from the other groups. CD was involved in the one discrepancy among methods that concerned the neglected group (no difference in likeability between average and neglected in Sample 1). NB and CD showed more difficulties to establish behavioral differences in Sample 1 (40% for both) than GB (47%).

### Research question 3: sources of disagreement between methods

In the method section we addressed the criteria and cutoffs used by each method as possible source of discrepancies in sociometric classifications. In the following paragraphs, we examine how the use of *z-*scores and composite dimensions in CD can affect the classification generated by this method, becoming the main sources of the differences with the probability methods.

GB does not use composite dimensions of PNR and NNR. NB uses composite dimensions only for the identification of neglected students, but as it uses absolute cutoffs previously established, the differences between the distributions of PNR and NNR do not affect the classification.

In contrast, the four specific criteria of CD are composite *z*-scores. CD assumes that the two composite dimensions are independent. But this is not so. We examined the association between impact and preference dimensions using multiple regressions, in which the dependent variable was alternatively one of the two dimensions, and the predictors were the other dimension, its square and its cube. The results indicate that SI is predicted by SP [significant curvilinear/quadratic estimator, *F*_(2, 223)_ = 51.642, *p* ≤ 0.001; *R*^2^ = 0.317, and SP is predicted by SI significant curvilinear/quadratic estimator, *F*_(2, 223)_ = 4.153, *p* = 0.012, *R*^2^ = 0.039].

The criterion also assumes that PNR and NNR contribute equally to exceeding the SP (SI) cutoff. But this is not so either. The distributions of both PNR and NNR are positively asymmetric (Gommans and Cillessen, 2015). However, the distributions of PNR and NNR are different: PNR displays more expansiveness than NNR (*M*_PNR_ = 2.79, range 2.33–3.00; *M*_NNR_ = 2.39, range 1.41–3.00), NNR displays more variance than PNR (Mean of *SD*_PNR_ = 2.20, range 1.44–2.94; Mean of *SD*_NNR_ = 2.82, range 1.54–4.8), but the variance coefficient (*VC*) is smaller for PNR than for NNR (Mean of *VC*_PNR_ = 0.79, range 0.55–1.06; Mean of *VC*_NNR_ = 1.18, range 0.69–1.82). The *VC* is particularly useful to compare two distributions that have a different mean. NPR and NNR meet the assumptions for the use of the *VC* (Escobar, 1998): there are only positive values with a lower limit of zero 0 and an upper limit of N-1, and N is relatively small.

Not only are the distributions of PNR and NNR different, but they also contribute differently to exceeding the cutoff of SP (or SI).In short, each distribution of PNR and NNR in each classroom has a *VC* and a *M*. First, as regards *VC*, we observe that every *VC* is associated with a single Z_0._ Z_0_ indicates the lowest value of the standardized distribution, that is, the *z-*score when PNR = 0 (written Z_PNR = 0_) or when NNR = 0 (written Z_NNR = 0_). We can notice that the smaller *VC* is, the larger |Z_0_| is. The larger |Z_0_|, the more it makes each nomination contribute more to exceeding the cutoff, since a larger |Z_0_| is more distant from the *M*. We refer to absolute values because the rules are valid for both PNR and NNR regardless of whether they are above or below the mean. Second, as regards the mean (*M*), we observe that the values of *M* and |Z_0_| determine the size of the interval between a score Z_i_ and the next Z_i+1_ (|Z_i_-Z_i+1_|), that is, the rate at which each new nomination increases Z_i_ (or decreases with each not-received nomination). A larger *M* makes the interval between Z_i_ and the next Z_i+1_ larger. In summary, the nominations belonging to distributions with smaller *VC* and larger *M* contribute more to exceeding the cutoff, and in all circumstances the contribution of *VC* is more significant than that of *M*. Therefore, we can say that in general PNR contribute more than NNR to exceeding the cutoff, since the *M* of PNR are larger and their *VC* are smaller than the ones of NNR.

We illustrate the before-explained with the following example. In one classroom X, the cutoff to be preferred or rejected is |Z_SP_| > 1, which is the same as ± 1*SD*_SP_. In this classroom, *SD*_SP_ = 1.59, the interval |Z_i_-Z_i+1_| is 45 for PNR (*M* = 3, *VC* = 0.74, Z_PNR = 0_ = −1.35) and 28 for NNR (*M* = 3, *VC* = 1.22, Z_NNR = 0_ = −0.82). In this classroom, a pair 5-0 is sufficient to exceed the cutoff to be preferred, |Z_PNR = 5_-Z_NNR = 0_| = |(0.9)–(−0.82)| = *1.72* > 1.59, and a pair 0-4 exceeds the cutoff to be rejected, |Z_PNR = 0_-Z_NNR = 4_| = |(−1.35)–(0.3)| = *1.65* > 1.59. We will now compare these two *pairs* (5-0; 0-4) to two other *pairs* that have a nomination more in both PNR and NNR (6-1;1-5), so that the differences between PNR and NNR remain the same (*SP* = 5 in the first *pair* and *SP* = 4 in the second *pair*). The fact that the PNR have greater homogeneity (small *VC*) and expansiveness (large *M*) than the NNR explains why a student is more likely to be preferred with a pair 6-1, for example, than with a pair 5-0, (|Z_PNR = 6_−Z_NNR = 1_| = |(1.35)–(−0.54)| = *1.89*, which is larger than 1.72 obtained with *pair* 5-0), whereas a student is more likely to be rejected with a *pair* 0-4, for example, than with a pair 1-5 (|Z_PNR = 1_−Z_NNR = 5_| = |(−0.90)–(0.58)| = *1.38* which is smaller than 1.65 obtained with *pair* 0-4). These examples show how PNR has more weight than NNR in determining both the preferred type (the more PNR a student receives, the more likely is he to be preferred) and the rejected type (the less PNR, the more likely be rejected). The fact that the NNR contribute less to exceeding the cutoff fosters the identification of rejected with few NNR. This also explains why the percentage of rejected is generally higher than the percentage of preferred when the CD method is used, because more PNR are required to reach the cutoff for preferred than NNR required to reach the cutoff for rejected.

### Research question 4: discrepant cases

We examined the sources of the discrepancy between procedures in the preceding subsections and in the method section. In the following paragraphs, we present the cases in which we observed discrepancy between methods, and we indicate afterward whether these differences in classification are supported by behavioral characteristics.

#### Comparison between standardized and probability methods (CD vs. NB and GB)

With regard to preferred, rejected, and controversial students, the discrepancies between CD and the other two methods were caused by the application of the criteria, but above all they stem from the use of *z*-scores, the distribution of nominations PNR and NNR, and the different contribution of the PNR and NNR in the composite dimensions (PNR+NNR; NPR-NNR).

CD identified as preferred, rejected, and controversial students with low values in PNR or NNR or in both. Of the 21 preferred with low values in PNR (with *pairs 5*-0, *5*-1, *4*-0), 19 were identified only by the CD procedure. Of the 19 rejected with low values in NNR (with *pairs* 0-*4*, 0-*3*, 1-*4*; 1-*3*; 0-*2*), 15 were identified only by the CD procedure. Ten students with *pairs* with low values were identified as controversial only by CD (5-3, 4-4, 4-2, 3-3, 3-5, 4-5, 5-5). These discrepancies can be explained by the fact that in CD it is usually enough that PNR for preferred or NNR for rejected be higher than *M*, while NB and GB are more restrictive and require that PNR or NNR be equal or higher than an upper limit.

In contrast, CD did not classify five participants with a high number of PNR or NNR as preferred or rejected. Three participants with a *pair* of *6*-2 or *6*-1 were not classified by CD as preferred. Two participants with a *pair* of 2-*7* were not classified by CD as rejected, whereas in the same classrooms participants with a *pair* 0-*5* were identified as rejected, which is an example of the higher influence of PNR (the fewer PNR) in the identification of rejected students.

Finally, we observed that CD categorized differently participants with the same *pair* PNR-NNR in different classrooms (intra-method variability). We found intra-method variability in 35 students with *pairs* 6-1, 5-0, 6-2, and 5-1, who were sometimes classified as preferred and sometimes not, and in 10 students with *pair* 2-5, who were sometimes classified as rejected and sometimes not. This variability was infrequent in GB and absent in NB.

We conducted one-way ANOVAs in order to know whether students classified differently by one or more procedures (inconsistent cases) differed behaviorally from those who had been classified the same by the three methods (consistent cases). For preferred, the inconsistent cases scored lower in likeability than the consistent cases, *F*_(1, 78_) = 5.023, *p* = 0.028, especially in Sample 2. For the rejected group, the inconsistent cases were less aggressive and less isolated than the consistent cases, *F*_(1, 91)_ = 4,442, *p* = 0.038, and *F*_(1, 91)_ = 4,816, *p* = 0.028, especially in Sample 1. There were no differences between the consistent preferred and rejected cases by the probability methods and CD. For the controversial group, inconsistent cases were less aggressive than consistent cases, *F*_(1, 31)_ = 5.382, *p* = 0.027, especially in Sample 1. In Sample 1, cases that were identified consistently by the two probability methods differed from those identified by CD, *F*_(1, 7)_ = 7.071, *p* = 0.033.

For neglected students, the inconsistent cases were as follows. CD identified students with *pair* 2-1 as neglected, which did not occur in the other procedures. As we may recall, CD, like NB, gives the same weight to PNR as to NNR, which makes them mere indicators of low impact without preference playing a role (SI ≤ 2 in NB, SI ≤ 3 in CD). GB, contrary to CD, did not allow the *pairs* 2-1 and 2-0. Regarding intra-method variability, we found also 87 cases in CD: with *pair* 2-1 (identified only 4 out of 22), *pair* 2-0 (15 out of 41), and *pair*1-2 (4 out of 14), and there were even three cases with *pair* 1-1 that were not identified as neglected. On the contrary, GB had variability only in 3 cases with *pair* 1-2 (14 out of 17), while NB, using an absolute cutoff, did not have any variation.

We ran one-way ANOVAs to compare consistent neglected (pairs 1-1, 1-0, 0-0, 0-1, 0-2) to inconsistent groups (pairs 2-1, 2-0, 1-2). Inconsistent neglected cases scored higher in likeability than consistent cases, *F*_(3, 113)_ = 3.426, *p* = 0.020. In the *post-hoc* multiple comparisons, we selected Tukey's HSD test when the data met the homogeneity of variances assumption, and Games Howell test when they did not*. Post-hoc* tests showed a significant difference between consistent and *pair* 2-0 (*p* = 0.025). Besides, there are four other arguments against classifying students with pair 2-0 as neglected. First, PNR = 2 is very close to *M*_PNR_. Second, more than 20% of students across samples had a value PNR = 2. Third, 7.5% of the sample had a *pair* 2-0. Fourth, 44% of neglected students with a *pair* 2-0 had positive reciprocities, against 17.6% of the remaining neglected students.

#### Comparison between probability methods (NB vs. GB)

With regard to preferred, rejected, and controversial students, the discrepancies between GB and NB are mainly due to the application of a criterion of an absolute pre-established cutoff or a computed one. We questioned whether the strict limit imposed by NB to identify preferred, rejected, and controversial was realistic. The discrepancy between NB and GB included students identified by GB but not by NB with PNR = 6 or 5 (22 with PNR = 5; 2 with PNR = 5) or NNR = 6, 5 or 4 (15 with PNR = 6; the other 15 with NNR = 5 or 4). We run one-way ANOVAs to compare students who were classified by both methods to those with a low score in nominations, which were classified only by GB. There were no behavioral differences. One exception was in Sample 1 where rejected students with NNR = 5 were less withdrawn than those with 7 or more NNR, *F*_(1, 19)_ = 8.64; *p* = 0.008.

With regard to neglected students, discrepancies between NB and GB were as follows. First, the fundamental difference was that NB identified as neglected 41 students with a *pair* 2-0 that were not identified as such by GB. These 41 students represented 40% of all neglected student identified by NB. Second, GB identified 14 students with a *pair* 1-2 that were not identified as such by NB. We could argue in the same vein that NNR = 2 is very close to the *M*_NNR_, but we think that 2 NNR are not the same as 2 PNR, especially when they are paired with zero nominations in the other dimension. We run one-way ANOVAs to compare students who were classified as neglected by both methods to those classified only by NB (2-0) or GB (1-2). As expected, students identified as neglected only by NB were more likeable than students identified by both NB and GB in Sample 1, *F*_(1, 36)_ = 5.213, *p* = 0.028, and in Sample 2, *F*_(1, 59)_ = 5.393, *p* = 0.024, and less withdrawn in Sample 2, *F*_(1, 59)_ = 4.254, *p* = 0.044. On the contrary, there were no behavioral differences between consistent and inconsistent neglected cases identified by GB (1-2).

## Discussion

In this study, three methods to identify sociometric types in the classroom were analyzed, one standardized and two probabilistic. Regarding goal 1, agreement between methods was high, overall and for each type (Terry and Coie, 1991; Frederickson and Furnham, 1998), although the three methods agreed in less than 50% of the participants in any type. Regarding goal 2, the classifications of the three methods showed good behavioral validity, coinciding with earlier research (Newcomb et al., 1993; García Bacete, 2006; Hymel et al., 2010), but there were behavioral differences in all the types among the students who were identified by all methods (consistent cases) and those who were not (inconsistent cases). These differences showed that inconsistent cases were less prototypical: the preferred were less likeable, the rejected were less aggressive and withdrawn, the controversial were less aggressive, and the neglected were more likeable.

Thus, there were clear disagreements between methods, confirming the need to examine the origin of such disagreements (Goals 3 and 4). The analysis of the scores, criteria, cutoffs, and dimensions allowed us to identify different elements involved in determining which students were identified in each type for each method. GB performed well, its rates for all types were intermediate between CD and NB, it reached the highest overall agreements with the other methods, and above all reflected best the behavioral differences across all types. GB, compared to CD and NB, made decisions based on maximizing the differences between PNR and NNR. CD had the most difficulties in identifying the types in the sample of younger children and differentiating controversial from neglected students. CD included many a priori conditions (PNR, NNR, Z_PNR_, Z_NNR_, SP, SI, Z_SP_, Z_SI_) that increase the possibilities for error in sociometric identification. But these difficulties in CD arose directly from using *z-*scores and composite dimensions, which are the core issues for the disagreement between standardized and probability methods, as seen in the introduction section.

### Use of *Z*-score and composite dimensions (standardized vs. probability methods)

*Z*-scores are more difficult to understand than raw scores, and only should be applied to normally distributed scores, which is usually not the case for PNR and especially NNR (Gommans and Cillessen, 2015). Hence, *z*-scores are less intuitive and do not represent adequately the social network in a group (Newcomb and Bukowski, 1983). When *z*-scores are applied in such a case, students can be classified in different types based on small differences in SP or SI (Maassen et al., 2005). The use of *z*-scores causes that the identification of types depends more on the variance than on the number of preferences (Newcomb and Bukowski, 1983).

The use of composite dimensions is based on two assumptions. The first assumption is that SP and SI are independent. However, they have a curvilinear relationship (Bukowski et al., 2000), and SP is a better predictor of SI than SI is of SP.

The second assumption is that PNR and NNR (Z_PNR_ and Z_NNR_) contribute equally to reaching the cutoff (Coie et al., 1982). CD processes PNR and NNR on an equal basis although their distributions are different (generally PNR is more expansive than NNR and its *VC* is much smaller than that of NNR). Therefore, they do not contribute to the same degree to SP (or SI) and hence to classification. For example, a *pair* 6-2 and a *pair* 5-1 are equally preferred since the difference is +4 in both cases, but CD is more likely to identify the first *pair* as preferred than the second *pair*. Similarly, a *pair* 0-4 and a *pair* 1-5 are equally rejected (the difference is −4), however the 0-4 *pair* is more likely to be identified as rejected than the 1-5 *pair*. This illustrates that PNR outweighs NNR in the identification of preferred students (the more PNR, the easier identified) and of rejected students (the least PNR, the easier identified). This situation is similar for controversial and neglected students. Besides, the importance of a large difference between PNR and NNR, of one of them being very high or very low, and of them being different one from one another, as warranted in NB and GB, seems to be unclear in CD. CD proposes complementarity between both, so that a minimal difference between PNR and NNR is enough in the case of preferred and rejected students, and no difference is required for controversial and neglected students.

These deficits in construct validity (the independence of composite dimensions, uneven contributions of NPR and NNR to the cutoffs, and the lack of similarity between *z* and raw scores) argue for caution when using the CD procedure.

As a result of the lack of construct validity a number of biases appear by using CD: classification of roughly similar percentages of students in each type in all classrooms, identification of more rejected than preferred students, a different classification of students with the same *pair* of nominations in different classrooms, classification as preferred (rejected or controversial) of students with low scores in PNR or NNR, and an increase of the heterogeneity among scores in each type. These problems appear especially in groups that are socially well-integrated with low *SD* for PNR or NNR, low *M* for NNR or high *M* for PNR (Newcomb and Bukowski, 1983).

Finally, we might doubt about the convenience of using social impact. While the construct of SP and SI was a major milestone in the history of sociometric classification (Cillessen and Bukowski, 2000), it is the very concept of SP and SI that highlights the problems of CD, due to the fact that the subtraction (or addition) of two different dimensions is not fully justified (Cillessen, 2009).

Furthermore, if SP and SI are not independent and PNR and NNR do not equally contribute to the cutoff, the question arises whether SI is needed. We think it is not, and that it is a secondary dimension with respect to SP. First, because SP predicts SI better than vice versa. But above all because students nominate on the basis of their preferences. Sociometry is essentially a measure of preference, at least what the students report is their positive and negative preferences for certain peers. However, SI is a measure of the number of preferences or visibility (Asher and McDonald, 2009) and does not take into account the positive or negative valence of these preferences when identifying controversial and neglected students. In fact, the controversial group can be understood as a complement to the preferred and rejected groups. Controversial students are those who are rejected or preferred who have a score in the other dimension higher than the mean. In the case of neglected students, in CD and NB the most important is not to have few PNR and NNR, but to have little prominence (social salience, visibility). However, if we want to know the degree of visibility or prominence, measures of centrality (e.g., Cairns et al., 1996; García Bacete and Marande, 2013) or perceived popularity (e.g., Hymel et al., 2010) may be more useful.

### Identification of neglected students by NB and GB

One of our goals was to study the changes proposed by GB with respect to NB for the identification of neglected students. The cases for which there are disagreements between the two methods were well-defined. NB identified as neglected students with a *pair* 2-0 while GB did not. GB identified as neglected students with a *pair* 1-2, but NB did not. The results show that students with a *pair* 2-0 were more likeable than the consistent neglected, while no behavioral differences were found with the *pair* 1-2, indicating that the identification of neglected with a *pair* 2-0 was not adequate. This inadequate identification is all the more important because this *pair* amounted to 40% of the neglected students identified by NB (30% of the neglected identified by CD). These results indicate the necessity of differentiating between PNR and NNR in identifying neglected students as done by GB, and not only in the number of nominations.

Moreover, CD displays a high variability in *pairs* that are admitted for classification as neglected and shows intra-method variability too in many *pairs* classified as neglected. CD displays the only discrepancy between methods in which the neglected are involved; it did not differentiate likeability between average and neglected in Sample 1, thus supporting the GB proposal of limiting the number of PNR to 1. In conclusion, GB is more suitable for identifying neglected students than NB and CD.

### Developmental considerations

Agreement between methods was higher in the older sample than in the younger sample. There were more behavioral differences between types in the older sample than in the younger sample. The results show that in older children the sociometric categories were behaviorally more defined, and therefore the identification of different sociometric groups is easier. Behavioral differences between students classified by all methods and classified by only one or two methods varied as a function of type and age: in the older sample there were more discrepant preferred and neglected cases; in the younger sample there were more discrepant rejected and controversial cases. In older children, the discrepancies between methods in preference may reveal some confusion between popularity and preference, common at these ages (Hymel et al., 2010), while the greater ambivalence toward withdrawn children might explain the discrepancies in the identification of neglected youths. Regarding the discrepancies related to rejection in the younger children, it could be because negative feedback is less available at this age (Bellmore and Cillessen, 2003).

### Limitations and applications

The results of this study are valid when nominations are limited to three. The next step will be to extend this study to unlimited nominations. GB and CD provide criteria that apply to unlimited nominations (González and García Bacete, 2010). ten Brink (1985) extended NB to unlimited nominations, making it possible to compare the three methods for unlimited nominations.

To make these results applicable for users, such as researchers and teachers, González and García Bacete (2010) developed the Sociomet program that calculates the main sociometric values, and individual and group indices, determines statistical significance for these values and yields the identification of sociometric types according to the GB criteria. The adequate sociometric classification, and the numerous sociometric characteristics, such as positive and negative reciprocities, are useful tools for improving both routine actions in school (e.g., grouping students in classroom configuration and groups within the classroom to enhance cooperative learning) and the design and application of socio-emotional interventions (e.g., friendship learning, focus on at risk-students), as proposed by García Bacete et al. (2014).

## Conclusion

Our results did not support the main assumptions used by standardized CD method. The CD method does not provide a frame of reference, it is a complex conceptual and computational procedure, and it is contrary to the intuitive idea of sociometric types. This may discourage its potential users (researchers and practitioners) and makes CD less recommended, especially for younger children, for studying long-term development, or for evaluating the effects of an intervention, because it does not reflect changes in context or voter population over time (Maassen et al., 2005).

As for the probabilistic methods, in this study we presented the GB method as an adaptation of the NB method. The results showed that the criteria proposed by GB for identifying neglected students were more suitable than the NB criteria (and CD criteria). This is an important contribution for the classification of neglected students, since it is the most difficult group to identify (Coie et al., 1982; Newcomb and Bukowski, 1983). In addition, GB has further advantages over NB. First, GB controls for the effects of non-voters. Second, its cutoffs are computed for each classroom, whereas NB uses absolute cutoffs only valid in situations of maximum expansiveness. Third, with regard to the identification of preferred, rejected, and controversial students, the strict criteria proposed by NB do not have behavioral justification. With regard to intervention, it is preferable to classify a few children at risk who may not be, than to not identify a few children who actually need help. In conclusion, the GB method yielded the most valid sociometric classifications across groups and ages.

## Author contributions

FG: Led and designed the study, coordinated data collection, performed the analysis and interpretation of the data, and drafted the manuscript. AC: Contributed to the analysis and interpretation of the data, revised critically the study, and participated in drafting and editing the manuscript. All authors approved the final manuscript as submitted.

### Conflict of interest statement

The authors declare that the research was conducted in the absence of any commercial or financial relationships that could be construed as a potential conflict of interest.
